# COLONOMICS - integrative omics data of one hundred paired normal-tumoral samples from colon cancer patients

**DOI:** 10.1038/s41597-022-01697-5

**Published:** 2022-10-01

**Authors:** Anna Díez-Villanueva, Rebeca Sanz-Pamplona, Xavier Solé, David Cordero, Marta Crous-Bou, Elisabet Guinó, Adriana Lopez-Doriga, Antoni Berenguer, Susanna Aussó, Laia Paré-Brunet, Mireia Obón-Santacana, Ferran Moratalla-Navarro, Ramon Salazar, Xavier Sanjuan, Cristina Santos, Sebastiano Biondo, Virginia Diez-Obrero, Ainhoa Garcia-Serrano, Maria Henar Alonso, Robert Carreras-Torres, Adria Closa, Víctor Moreno

**Affiliations:** 1 Oncology Data Analytics Program, Catalan Institute of Oncology (ICO). Hospitalet de Llobregat, Barcelona, Spain; 2 Colorectal Cancer Group, ONCOBELL, Bellvitge Biomedical Research Institute (IDIBELL). Hospitalet de Llobregat, Barcelona, Spain; 3 Biomedical Research Centre Network for Epidemiology and Public Health (CIBERESP), Madrid, Spain; 4 Molecular Biology CORE, Center for Biomedical Diagnostics, Hospital Clínic de Barcelona, 08036 Barcelona, Spain; 5 Translational Genomic and Targeted Therapeutics in Solid Tumors, August Pi i Sunyer Biomedical Research Institute (IDIBAPS), 08036 Barcelona, Spain; 6 Unit of Nutrition and Cancer, Cancer Epidemiology Research Program, Catalan Institute of Oncology (ICO) - Bellvitge Biomedical Research Institute (IDIBELL). L’Hospitalet de Llobregat, Barcelona, 08908 Spain; 7 Department of Epidemiology, Harvard T.H. Chan School of Public Health, Boston, MA 02115 USA; 8 Rheumatology Department - Parc Taulí Research and Innovation Institute (I3PT), Barcelona, Spain; 9 TIC Salut Social Foundation. Ministry of Health of Generalitat de Catalunya, Barcelona, Spain; 10 Reveal Genomics. S.L., Barcelona, Spain; 11 Department of Clinical Sciences, Faculty of Medicine and health Sciences and Universitat de Barcelona Institute of Complex Systems (UBICS), University of Barcelona, Barcelona, Spain; 12 Medical Oncology Department. Catalan Institute of Oncology (ICO), Hospitalet de Llobregat, Barcelona, Spain; 13 Biomedical Research Centre Network for Oncology (CIBERONC), Madrid, Spain; 14 Pathology Service, Bellvitge University Hospital (HUB), Hospitalet de Llobregat, Barcelona, Spain; 15 Digestive Surgery Service, Bellvitge University Hospital (HUB). Hospitalet de Llobregat, Barcelona, Spain; 16 The John Curtin School of Medical Research, Australian National University, Canberra, Australia; 17 EMBL Australia Partner Laboratory Network at the Australian National University, Canberra, Australia

**Keywords:** Cancer genomics, Biomarkers, Colon cancer

## Abstract

Colonomics is a multi-omics dataset that includes 250 samples: 50 samples from healthy colon mucosa donors and 100 paired samples from colon cancer patients (tumor/adjacent). From these samples, Colonomics project includes data from genotyping, DNA methylation, gene expression, whole exome sequencing and micro-RNAs (miRNAs) expression. It also includes data from copy number variation (CNV) from tumoral samples. In addition, clinical data from all these samples is available. The aims of the project were to explore and integrate these datasets to describe colon cancer at molecular level and to compare normal and tumoral tissues. Also, to improve screening by finding biomarkers for the diagnosis and prognosis of colon cancer. This project has its own website including four browsers allowing users to explore Colonomics datasets. Since generated data could be reuse for the scientific community for exploratory or validation purposes, here we describe omics datasets included in the Colonomics project as well as results from multi-omics layers integration.

## Abstract (summary)

**Table:** 

Measurement(s)	Colon Gene expression • Colon DNA methylation • Colon Genptyping data • Colon Copy Number Variation • Colon miRNAs • Colon Whole Exome Sequencing
Technology Type(s)	Affymetrix Human Genome U219 Array Plate platform • Illumina Infinium HumanMethylation 450k BeadChip • Affymetrix Genome-Wide Human SNP 6.0 array • Small RNA Assay of the Agilent 2100 Bioanalyzer • Agilent kit Sure Select XT Human All Exon 50MB
Factor Type(s)	batch • background
Sample Characteristic - Organism	Homo sapiens
Sample Characteristic - Environment	colonic mucosa
Sample Characteristic - Location	Catalonia Autonomous Community, Spain

## Background & Summary

**Fig. 1:**
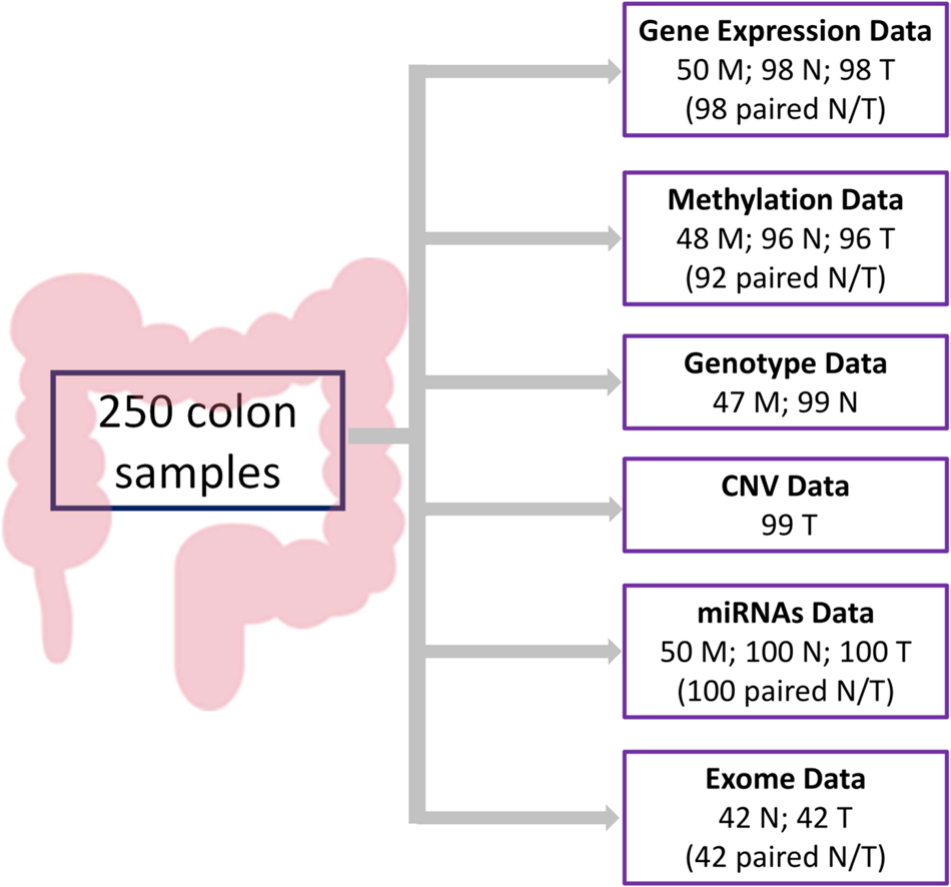
Scheme of Colonomics data. Number of samples that remained after quality control for each type of data. M are healthy colon mucosae, T is tumoral tissue and N is normal tissue adjacent to tumor.

Colon cancer is a complex disease characterized by an accumulation of alterations at different molecular levels. Many studies focus on one molecular level, leading to a partial view of the carcinogenic process. Here we present Colonomics (https://www.colonomics.org/), a multi-omics dataset that comprises 100 stage II colon cancer patients from which samples of tumoral tissue (T) and paired normal adjacent tissue (N) were obtained from the surgical specimen. Also, 50 samples from healthy colon mucosa obtained at colonoscopy from donors (M) without cancer were included and used as reference (Fig. 1).

Multiomics data analysis allows to integrate different levels of biological and molecular information to understand complex diseases as cancer and to move forward towards precision medicine^1,2^. The Colonomics project aims to study the molecular basis of colon cancer through the integrative and large-scale analysis of different molecular data and to identify useful biomarkers of diagnosis and prognosis in colon cancer.

From each sample, DNA and RNA were extracted to obtain molecular data. Thus, extracted DNA was used to acquire methylation data (using Illumina microarrays), single nucleotide polymorphism (SNP) genotypes and CNV (using Affymetrix microarrays) and somatic mutations (through whole exome sequencing) in a subset. RNA was used to obtain gene and miRNAs expression, using Affymetrix microarrays and SOLID sequencing, respectively. Finally, patient clinical data from all samples were obtained, including follow up.

The analysis of these datasets allowed us to make a descriptive and a comparative snapshot of the molecular state of cancer cells (T) and, also, to find differences between healthy normal tissue (M), normal tissue adjacent to tumor (N), and tumoral tissue (T).

The Colonomics project web page (https://www.colonomics.org/) links to the digital data repository of the University of Barcelona (UB-DD)^3^ and provides four user friendly web tools that allow an agile and direct visualization of data.

## Methods

### Patients and samples

Colon cancer patients were retrospectively selected during 2012 from those that had received radical surgery in the Bellvitge University Hospital (Barcelona, Spain) between January 1996 and December 2000, and had fresh frozen tumor samples in the hospital biobank. One hundred patients were included, with the criteria of a confirmed adenocarcinoma of the colon, diagnosed in stage II and the tumor was microsatellite stable (tested with five markers). Patients should have not received adjuvant therapy and a minimum follow up of three years was requested at the time of selection. A pathologist reviewed the tumor blocks, confirming diagnosis and that there were no signs of tumor cells when margins were examined. The paired adjacent normal mucosa (N) was obtained from the proximal margin, at least 10 cm distant from the tumor (T). As a control group, 50 healthy mucosa donors (M) were prospectively invited to participate in this study when they underwent a colonoscopy indicated for screening or symptoms with no evidence of lesions in the colon or rectum. Table 1 shows the basic clinical characteristics of the samples. The study protocol was approved by The Clinical Research Ethics Committee (CEIC) of the Bellvitge Hospital. All the recruited individuals provided a written informed consent to participate in the genetic study. The approval number is PR178/11.

**Table 1:** Clinical characteristics of the 250 samples.

	n	Female	Male	Age min	Age 1Q	Age median	Age mean	Age 3Q	Age max	Colon Site Left	Colon Site Right
M	50	23	27	25	52	63	62.5	74	88	23	27
N	100	28	72	43	65	71.5	70.7	78	87	61	39
T	100	28	72	43	65	71.5	70.7	78	87	61	39
All	250	79	171	25	64	70	69.0	77	88	145	105

M is healthy colon mucosae, T is tumoral tissue and N is normal tissue adjacent to tumor.

From these 250 samples (100 T, 100 N and 50 M), data on gene expression, DNA methylation, SNPs, CNV and miRNAs were obtained. Whole exome sequencing was analyzed in a subset of 42 tumors and their paired adjacent normal mucosa. Figure 1 shows the number of samples for each type of data.

### DNA extraction

DNA was extracted from colon tissue by the phenol-chloroform procedure, quantified using a NanoDrop ND 2000c spectrophotometer (NanoDrop Thermo Scientific, DE, USA) and stored at 4 °C. Bisulphite conversion of DNA (200–500 ng) was performed according to the manufacturer’s recommendations for the Illumina Infinium Assay (EZ DNA methylation kit. Zymo Research. Cat. No. D5004). The incubation profile was 16 cycles at 95 °C for 30 seconds, 50 °C for 60 minutes and a final holding step at 4 °C.

### RNA isolation

Total RNA was isolated from tissue samples using Exiqon’s miRCURY™ RNA Isolation Kit (Exiqon, Denmark). For quantification, NanoDrop® ND-1000 Spectrophotometer (Nanodrop technologies, Wilmington, DE) was used. RNA quality was assessed by gel electrophoresis and RNA 6000 Nano Assay (Agilent Technologies, Santa Clara, CA). RNA purity was measured with the ratio of absorbance at 269/280 nm (mean = 1.96, sd = 0.04), no differences among tissue types were found. RNA integrity numbers showed good quality (mean = 8.1 for T, and 7.5 for N).

### Gene expression data

Affymetrix Human Genome U219 Array Plate platform (Affymetrix, Santa Clara, CA, USA) was used to obtain gene expression data from isolated RNA. A block experimental design was performed to the 96-array plates to avoid batch effects and sample ids were blinded to laboratory technicians. Robust Multiarray Average (RMA) algorithm in *affy* package (version 1.28) from R/Bioconductor^4^ was used to normalize data. After quality control and normalization 49,386 probesets in 20,070 genes and 246 samples remain for the analysis: 50 M, 98 N and 98 T (98 paired N/T). Since the array provides multiple probes per gene, a summary gene expression value for each gene was obtained through a principal components analysis (PCA). We extracted the first principal component, that captures the highest expression variability, and was rescaled to have the average expression of the different proves and maximum observed standard deviation. This rescaling was required to avoid negative expression values of the first PC. Therefore, in addition of the dataset with the measured probes, a final dataset with a single expression value for each one of the 20,070 genes was obtained.

### Methylation data

The Illumina Infinium HumanMethylation 450k BeadChip assay was used to obtain the DNA methylation profile of the samples. The array interrogates the methylation levels of 485,512 CpG sites covering 99% of RefSeq genes and 96% of CpG islands^5,6^. To minimize batch effects, samples were randomly distributed but matching paired samples (N, T) in the same array row (M samples were randomly paired in array rows).

Sample quality and sex concordance was checked using the SNPs of the 450 K array with those of the Affymetrix Genome-Wide Human SNP 6.0 array (Affymetrix, Santa Clara, USA). A total of 10 samples had to be excluded due to quality problems and the final dataset contained 240 samples: 96 N, 96 T (92 complete pairs of N/T) and 48 M.

Background correction was performed filtering out probes with a detection p-value greater than 0.01 in more than 5% of the samples. We discarded probes that ambiguously mapped to multiple locations in the human genome with up to two mismatches^7^. We also excluded probes that contained SNPs within 10 bp of distance. Finally, a set of 430,086 probes was obtained.

We used β-values, the Illumina’s standard, to measure methylation level at each locus. β-values are calculated using the average probe intensity and they range from 0 to 1, being β = 0 the absence of methylation and β = 1 a complete methylation. Subset-quantile within array normalization (SWAN) was used to reduce technical variation within and between arrays^8^ using the Bioconductor package *minfi* (version 3.0.1)^9^. A random forest approach was used for the imputation of missing values.

### Genotyping data

Extracted DNA was hybridized to obtain the genotypes of the samples using Affymetrix Genome-Wide Human SNP 6.0 array (Affymetrix, Santa Clara, USA), which includes near 1 million of SNP markers. Genotype calling was performed with Corrected Robust Linear Model with Maximum Likelihood Classification (CRLMM) algorithm as implemented in R/Bioconductor package *crlmm* (version 1.8)^10^. A total of 4 samples were excluded due to quality or sex concordance problems (3 M and 1 N) so 146 samples (47 M and 99 N) remained for the analysis.

### Copy number variation data

The Affymetrix Genome-Wide Human SNP 6.0 genotyping array was used to infer the copy number aberrations in the tumors. The average inter-marker distance of this array was 700 bp. Copy number estimate (CNE) was calculated using Affymetrix Power Tools (Version 1.16.1) software^11^ with default parameters.

Adjacent locus with a similar CNE were grouped in regions using a segmentation pipeline that had three steps. First, a smoothing spline was applied to normalize data. Second, change points in CNE pattern were located using the Vega R package (version 1.35)^12^ and split into discrete segments. To avoid masking effects, the stromal content of the tumor samples was taken into account to calculate the thresholds to split the segments. Stromal content was estimated using the *ESTIMATE* R package (version 1.0.13)^13^ using gene expression data^14^. A hierarchical cluster analysis was performed to divide the samples in 4 clusters reflecting their different levels of stromal content. A different threshold to split the segments was used for each cluster: ±0.5 for low stroma, ±0.4 for medium-low stroma, ±0.3 for medium-high stroma and ±0.2 for high stroma^15^. The third and final step of the segmentation process consisted in a t-test to compare the mean of consecutive segments and to merge them in case there are no statistical differences (p-value > 1e-04). For each segment, the mean of CNE was assigned as the representative value of the region. 26,423 segments of CNV were found in the 99 T samples (15,646 losses and 10,777 gains).

### miRNA expression data

Isolated RNA was used to obtain the expression of miRNAs. Small RNA Assay of the Agilent 2100 Bioanalyzer (Agilent Technologies, Santa Clara, CA) was used to assess the quality control of the miRNA fraction following manufacturer’s recommendation. Small RNA-seq was performed through the SOLiD platform. The PureLink miRNA isolation kit was used to construct the libraries of compatible fragments with SOLiD from an enriched fraction of small RNA. Sequencing microspheres were obtained by applying an emulsion PCR into an equimolar mixture of 48 libraries followed by an enrichment process before charging in the reaction chamber. Finally, the reaction to obtain the sequences (of 35nt + 10nt barcode) from miRNA fraction was performed by blinded laboratory technicians through the Applied Biosystems SOLiD 4 System. The samples were randomly distributed among the different sequencing slides to minimize batch effects. The data quality was estimated using the SOLiD Experimental Tracking System (SETS) software. Parameters such as total number of reads, proportion of reads with miscalls or proportion of reads with low average quality value (QV) were evaluated for quality control with HTS SOLiD Preprocessing software^16^, removing reads containing a miscall as well as reads containing negative quality scores. All 250 samples showed adequate quality. Quantification of specific miRNAs was performed mapping the reads to the reference of mature miRNA sequences annotated in miRBase release 22.1 (October 2018) that contained 2,654 human miRNA sequences^17^. The FASTX-toolkit^18^ was used to preprocess the miRNA data and to provide compatible sequences for mapping with the Bowtie 1.2.2 aligner^19^. Cutadapt^20^ software was used to trim read adapter and the final table of counts was generated with SAMtools mpileup v1.8^21^. Finally, 2,641 miRNAs remained for the analysis.

### Whole exome sequencing

Genomic DNA from a subset of 42 N and T paired samples was sequenced in the National Center of Genomic Analysis (Barcelona, Spain; CNAG) using the Illumina HiSeq- 2000 platform. Samples selection was required due to budgetary constraints. Samples included were those from all patients that had experienced tumor progression (n = 21), and a matched sample from a patient with more than 5 years of follow-up without progression.

Exome capture was performed with the commercial Agilent kit Sure Select XT Human All Exon 50MB. Exomes from T were sequenced at 60x coverage (2 × 75 bp reads), and exomes from N were sequenced at 40x (2 × 75 bp reads). The sequences were quality controlled using FastQC software^22^ and aligned over the human genome hg19 version, with Bowtie 2.0^23^. Unmapped reads, reads with unmapped mate, nonprimary alignments, and reads that were PCR or optical duplicates were discarded with Picard^24^. A local realignment around indels found in our samples and defined in dbSNP 135.b37 version^25^ and 1000 genomes project^26^ was applied. Variant calling was performed with GATK software^27^, and low-quality variants (mapping quality below 30, read depth below 10 or frequency <10%) were filtered out. Germline variants from our study that are the variants found in normal adjacent paired tissue but not in tumor were removed. Other germline variants found in normal tissue from 1000 genomes project were also filtered out. Only single-nucleotide variants (SNV) were included in this study. Finally, variants were annotated using the SeattleSeq Variant Annotation web tool^28^. No correlation was observed between the number of mutations per sample and the control quality parameters (number of reads, number of no matched reads, percentage of unique aligned reads, number of duplicate reads and coverage). Finally, 13,015 somatic variants (11,126 SNVs) were found.

## Data Records

All the Colonomics data is publicly available, though it has been deposited in diverse repositories. Table 2 summarizes the accession for the different types of data. To facilitate performing multiomics analysis, downloading and merging the different sets, all samples have the same identifiers in all the upload datasets. We provide the processed data in the Colonomics website (https://www.colonomics.org/), and in the digital data repository of the University of Barcelona (UB-DD)^3^, to ensure permanent availability.

**Table 2:** Data accession summary table.

	Data	Format	Elements	N samples		Accession	Accession 2
Expression	raw data	CEL files	49,534 probes	246	GEO	https://identifiers.org/geo:GSE44076	—
	normalized data (log2 RMA)	txt files	49,386 probes	246	GEO/UB-DD	https://identifiers.org/geo:GSE44076	10.34810/DATA169
	summary by gene (PC1)	txt file	20,070 genes	246	UB-DD	10.34810/DATA169	
Methylation	raw data	idat files	485,512 CpGs	240	GEO	https://identifiers.org/geo:GSE131013	—
	probes annotations	txt file	430,086 CpGs	—	UB-DD	10.34810/DATA169	—
	normalized data (betas)	txt file	430,086 CpGs	240	GEO/UB-DD	https://identifiers.org/geo:GSE131013	10.34810/DATA169
SNPs	raw data	CEL files	1 M SNPs	146	EGA	https://ega-archive.org/datasets/EGAD00010001253	—
CNV	segment data	txt file	54,002 segments	99	UB-DD	10.34810/DATA169	—
miRNAs	raw data	fastq file	probes	250	EGA	https://ega-archive.org/datasets/EGAD00001004827	—
	count data	txt file	2,641 miRNAs	250	UB-DD	10.34810/DATA169	—
Whole Exome	raw data	fastq files (2 x sample)	reads	84	EGA	https://ega-archive.org/datasets/EGAD00001004826	—
Somatic Mutations	mutation data	txt file	13,015 mutations	42	UB-DD	10.34810/DATA169	—
Clinical data	clinical data	txt file	covariates	250	UB-DD	10.34810/DATA169	—
Expression Predictive Models	predictive model	txt/db files	gene/SNP info	—	ZENODO	10.5281/zenodo.6334768	—
Methylation Predictive Models	predictive model	txt/db files	CpG/SNP info	—	ZENODO	10.5281/zenodo.6334768	—
miRNAs Predictive Models	predictive model	txt/db files	miRNA/SNP info	—	ZENODO	10.5281/zenodo.6334768	—
MultiAssayCLX.Rdata	MultiAssayExperiment R object	Rdata	49,534 expression probes/430,086 methylation CpGs/2,641 miRNAs/38,905 CNV segments/Clinical data	—	UB-DD	10.34810/DATA169	—

Gene expression includes 246 samples: 50 M, 98 N and 98 T. Gene expression raw and normalized data are available in the Gene Expression Omnibus database (GEO) through BioProject PRJNA188510 and accession number GSE44076^29^. Raw data was upload as 246 CEL files and the normalized data is included within sample table as text file. The normalized data as log2 RMA signals include 49,386 probesets and can also be downloaded from UB-DD^3^. Finally, the gene expression summarized at gene level is available at the UB-DD^3^ and includes 20,070 genes.

DNA methylation data, both, raw and normalized are also available in GEO through BioProject PRJNA542323 and accession number GSE131013^30^. Methylation data includes 240 samples: 48 M, 96 N and 96 T. We provide 240 IDAT files that correspond to raw data and a text file with the normalized beta values of the 240 samples and 430,086 CpG probes. The normalized methylation data is also available at the UB-DD^3^ together with the annotation of the 430,086 CpG methylation probes. Both gene expression and DNA methylation data are part of the GEO super series GSE166427^31^.

Raw genotyped SNP data can be obtained, under controlled access, at the European Genome-phenome Archive (EGA) through accession number EGAD00010001253^32^. It includes 146 CEL files that corresponds to 47 M and 99 patients of colon cancer.

The processed CNV data of 99 T samples is accessible in the UB-DD^3^. It includes 54,002 segments: 15,646 losses, 10,777 gains and 27,579 segments that are not gains nor losses.

Raw small RNA sequencing data is also available under request in EGA through accession number EGAD00001004827^33^. We provide 250 fastq files, one for each sample (50 M, 100 N and 100 T). Count data of the 2,641 miRNAs and the 250 samples are accessible at UB-DD^3^.

Raw whole exome sequencing data of 84 samples (42 N and 42 T) with 2 fastq files per sample are accessible in EGA under request through accession number EGAD00001004826^34^. The tumors mutations identified are available at UB-DD^3^.

SNPs, miRNAs and whole exome data are in the EGA repository under the Data Access Committee through accession number GAC00001000662^35^.

Finally, somatic mutations of the 42 samples can be downloaded from UB-DD^3^. 13,015 somatic variants were included.

In addition to raw and preprocess multiomics data, clinical information of the samples is also upload at UB-DD^3^. This file is txt formatted and contains a row for each sample and a column for each variable. The variables included are: *id_clx*: the identifier of the Colonomics sample; *type*: tissue type (Mucosa, Normal or Tumor); *id_clx_individual*: the individual identifier; *stage*: the stage of the tumor (IIA or IIB); *sex*: (Female or Male); *age*; *site*: the site of the colon (Left/Right); *event_free*: a binary variable that indicates if the patient has a recurrence of colon cancer (1) or not (0); *time_free*: a continuous variable that indicates the time from surgery to recurrence of colon cancer; *event_global*: a binary variable that indicates if the patient is still alive (1) or not (0); *time_global*: a continuous variable that indicates the time from surgery to death; *metastasis_site*: the organ where a metastasis has appear; *BRAF_mutated*: a binary variable that indicates if the tumor is BRAF mutated (Yes) or not (No); *KRAS_mutated*: a binary variable that indicates if the tumor is KRAS mutated (Yes) or not (No); *stromal_score*: stromal score to predict the level of infiltrating stromal cells in tumor samples obtained using ESTIMATE package (version 1.0.13)^13^ from R and *CMS*: consensus molecular subtype classification of the tumoral samples in 4 groups (CMS1, CMS2, CMS3 or CMS4) obtained using CMScaller package (version 0.1)^36^ from R and based on the paper of Guinney *et al*.^37^.

To facilitate working with all these data and to avoid downloading each dataset individually, a MultiAssayExperiment object from R (version 1.22)^38^ has also been created. This object includes clinical data, data from gene expression, methylation, miRNAs and copy number variation and it is accessible in UB-DD^3^.

In addition to clinical and multiomics data, gene expression prediction models from SNP data, useful for Transcriptome-Wide Association Studies (TWAS), can be download from Zenodo through accession number 6334768^39^. These models were built for normal colon tissue (samples N + M) using elastic net regression, following the PredictDB pipeline^40,41^. They include significant prediction for 1,758 genes, 17,281 CpG probes and 39 miRNAs. See the usage notes section for more details.

## Technical Validation

We performed technical validations and results replication in other datasets for some of the Colonomics assays. For gene expression, a selected group of genes were assessed with multiplexed RT-qPCR using BioMark Dynamic Array 96 × 96 Plates (Fluidigm Corporation, San Francisco, CA). *ACTB*, *TPT1*, and *UBC* were used as control genes in the assay, see Solé *et al*.^42^ for details. Also, differentially expressed genes were validated using 45 N/T paired samples from The Cancer Genome Atlas (TCGA) data^43^, obtaining that the 97.86% of our differentially expressed genes were also differentially expressed in TCGA data^44^. Differentially expressed genes were found using *limma* R package (version 3.42)^45^. Genes with an absolute log fold change >1 and a Bonferroni adjusted P-value < 0.05 were defined as differentially expressed.

Regarding DNA methylation data, the list of the differentially methylated CpGs obtained using Colonomics data was compared with the one obtained using the 45 N/T paired samples from TCGA data obtaining that the 99.96% of the common CpGs were also differentially methylated in TCGA data^44^. Also, CNV was validated using TCGA data. In this case, 222 colon cancer samples were used to compare the obtained 13,279 minimal recurrent regions and 66% of these regions were also found in TCGA data^15^.

In the analysis of whole exome sequencing data, 13 SNPs in the 84 samples were genotyped using KASPar genotyping assays (KASP-By-Design; LGC Group) on the Fluidigm genotyping platform (48.48 Dynamic Array IFG, Fluidigm). These data were used to assess concordance between samples. All 42 N samples correctly matched with their corresponding T sample^46^. Also, to validate recurrent mutations found in AMER1 gene, sanger sequencing was used. All mutations were validated.

As a validation of the discovery pipeline of SNVs, 6 mutations in KRAS (Q61H, A146T, G12V, G12D, G12S, G13D) and 7 in TP53 (G245D, R248Q, R237H, R273C, R175H, R282W, R213_, G245S) were tested using KASPar genotyping assays in the Fluidigm Biomark platform (dynamic arrays). A 65% of concordance were achieved. It is noteworthy that 10 out of 11 no concordant mutations were only found by exome sequencing thus confirming the higher sensitivity of this technique.

## Usage Notes

### Re-use of the data and study limitations

All the data in the Colonomics project has been analyzed and used in published papers except for miRNAs data. The first paper involving Colonomics data was published eight years ago in 2014. The data were analyzed with the best algorithms and programs at that time, but nowadays there may exist improved analysis pipelines. That’s why we have shared raw data for each omic layer in addition to preprocessed data. Specifically, for the case of whole exome sequencing data that was processed 8 years ago, we are aware that the calling algorithms may have produced some false positive mutations.

When re-using Colonomics data we also need to consider that all samples were confirmed adenocarcinoma of the colon, diagnosed in stage II and the tumor was microsatellite stable. Colonomics data is a very homogeneous cohort, but the absence of rectal tumors, colon tumors in other stages rather than stage II and microsatellite instable samples may limit building generalizable hypothesis of colorectal cancer. Patients were selected to have received only radical surgery, without adjuvant chemotherapy, which also reduces potential analysis to prognosis but not prediction.

### Web browsers

In order to visualize the different types of data, we developed different intuitive and user friendly shiny^47^ web applications that are freely accessible at https://www.colonomics.org/data-browser. There are four different web applications: the expression browser, the methylation browser, the expression quantitative trait loci (eQTL) browser and the regulatory networks browser. In these browsers, expression, methylation, and genotyping data have been exploited developing different types of analysis. A web browser integrating all the types of omics datasets available would be an interesting future application.

### Genetic prediction models for normal mucosal biopsies

The genetic prediction models represent reference imputation panels for normal colon tissue methylation and gene and miRNA expression, which are of high interest for performing TWAS studies for colon-related diseases.

We provided significant prediction models for 1,758 genes, 17,281 CpG probes and 39 miRNAs obtained from normal biopsy samples. These features can be predicted from SNPs located within ± 1 Mb, which we assumed they act through cis mechanisms. We included the model’s summary statistics and corresponding SNP weights in SQLite objects^39^. Models were trained using the elastic net procedure employed in the PredictDB pipeline^40,41^, according to which only models with a predictive performance p-value < 0.05 and R^2^ >0.1 are considered significant. We adjusted the models by basic covariates, i.e., sex, age, tissue type and colon anatomic location where biopsies were collected (left and right colon). Genome coordinates refer to GRCh37/hg19.

Prediction models for gene expression were trained using 144 normal mucosal biopsy samples (97 N and 47 M), the common samples between gene expression and SNPs. We trained models for 13,939 genes (including protein coding, long non-coding and pseudogenes), which had more than 3 sequencing reads in more than 10% of the samples. In cases where multiple probes mapped to a single gene, the first principal component from PCA was used to capture the largest common variability. Trimmed Mean of M-values (TMM) normalization was applied. TMMs were transformed with inverse normal transformation.

Prediction models for CpG probes were trained using 132 normal mucosal biopsies (95 N and 37 M). Only CpG annotated in islands, shores and shelves were taken into account as CpGs in open-sea are not functionally interesting. We also filter in CpGs which had more than 3 sequencing reads in more than 5% of the samples. After these filters, 257,809 CpG remained for the analysis. Inverse normal transformation on Beta MIxture Quantile (BMIQ)-normalized values was applied. In addition to the basic covariates for adjustment, we adjusted the models by 10 probabilistic estimation of expression residuals (PEER) factors^48^ to capture additional technical variability.

Prediction models for miRNAs were trained using 146 normal mucosal biopsies (99 N and 47 M), the common samples between miRNAs and SNPs. From the 2,641 miRNAs, we included 739 miRNAs, which had more than 3 sequencing counts in more than 10% of the samples. TMM normalization was applied and transformed with inverse normal transformation. As SNP data is in hg19 genome built, annotations of miRNAs refer to the miRBase release 20^17,49^.

## Data Availability

Code used to process and analyze the data is available upon request. Some R scripts can be downloaded from https://github.com/odap-ico/colonomics.

## References

[1] Marshall JL (2022). The Essentials of Multiomics. The Oncologist.

[2] de Anda-Jáuregui G, Hernández-Lemus E (2020). Computational Oncology in the Multi-Omics Era: State of the Art. Front. Oncol..

[3] Moreno Aguado V, Sanz Pamplona R, Díez Villanueva A (2022). Repositori de Dades de Recerca.

[4] Gautier L, Cope L, Bolstad BM, Irizarry R (2004). A. affy–analysis of Affymetrix GeneChip data at the probe level. Bioinformatics.

[5] Bibikova M (2011). High density DNA methylation array with single CpG site resolution. Genomics.

[6] Bibikova M (2009). Genome-wide DNA methylation profiling using Infinium® assay. Epigenomics.

[7] Price ME (2013). Additional annotation enhances potential for biologically-relevant analysis of the Illumina Infinium HumanMethylation450 BeadChip array. Epigenetics & Chromatin.

[8] Maksimovic J, Gordon L, Oshlack A (2012). SWAN: Subset-quantile within array normalization for illumina infinium HumanMethylation450 BeadChips. Genome Biol..

[9] Aryee MJ (2014). Minfi: a flexible and comprehensive Bioconductor package for the analysis of Infinium DNA methylation microarrays. Bioinformatics.

[10] Scharpf RB, Irizarry RA, Ritchie ME, Carvalho B, Ruczinski I (2011). Using the R Package crlmm for Genotyping and Copy Number Estimation. J Stat Softw.

[11] Eckel-Passow JE, Atkinson EJ, Maharjan S, Kardia SL, de Andrade M (2011). Software comparison for evaluating genomic copy number variation for Affymetrix 6.0 SNP array platform. BMC Bioinformatics.

[12] Morganella S, Cerulo L, Viglietto G, Ceccarelli M (2010). VEGA: variational segmentation for copy number detection. Bioinformatics.

[13] Yoshihara, K., Kim, H., & Roel, G. W. Verhaak. estimate: Estimate of Stromal and Immune Cells in Malignant Tumor Tissues from Expression Data. *R package version 1.0.13/r21* (2016).

[14] Yoshihara K (2013). Inferring tumour purity and stromal and immune cell admixture from expression data. Nat Commun.

[15] Alonso MH (2017). Comprehensive analysis of copy number aberrations in microsatellite stable colon cancer in view of stromal component. Br J Cancer.

[16] Sasson A, Michael TP (2010). Filtering error from SOLiD Output. Bioinformatics.

[17] Kozomara A, Birgaoanu M, Griffiths-Jones S (2019). miRBase: from microRNA sequences to function. Nucleic Acids Research.

[18] Pearson WR, Wood T, Zhang Z, Miller W (1997). Comparison of DNA Sequences with Protein Sequences. Genomics.

[19] Langmead B, Trapnell C, Pop M, Salzberg SL (2009). Ultrafast and memory-efficient alignment of short DNA sequences to the human genome. Genome Biol.

[20] Martin M (2011). Cutadapt removes adapter sequences from high-throughput sequencing reads. EMBnet j..

[21] Li H (2009). The Sequence Alignment/Map format and SAMtools. Bioinformatics.

[22] Simon Andrews. *FastQC: A quality control tool for high throughput sequence data*.

[23] Langmead B, Salzberg SL (2012). Fast gapped-read alignment with Bowtie 2. Nat Methods.

[24] *Picard: A set of command line tools (in Java) for manipulating high-throughput sequencing (HTS) data and formats such as SAM/BAM/CRAM and VCF*.

[25] Sherry S (2001). T. dbSNP: the NCBI database of genetic variation. Nucleic Acids Research.

[26] Devuyst O (2015). The 1000 Genomes Project: Welcome to a New World. Perit Dial Int.

[27] DePristo MA (2011). A framework for variation discovery and genotyping using next-generation DNA sequencing data. Nat Genet.

[28] Ng SB (2009). Targeted capture and massively parallel sequencing of 12 human exomes. Nature.

[29] (2014). GEO.

[30] (2020). GEO.

[31] (2021). GEO.

[32] (2022). EGA.

[33] (2022). EGA.

[34] (2022). EGA.

[35] (2022). EGA.

[36] Eide PW, Bruun J, Lothe RA, Sveen A (2017). CMScaller: an R package for consensus molecular subtyping of colorectal cancer pre-clinical models. Sci Rep.

[37] Guinney J (2015). The consensus molecular subtypes of colorectal cancer. Nat Med.

[38] Ramos M (2017). Software for the Integration of Multiomics Experiments in Bioconductor. Cancer Research.

[39] Moreno V, Diez-Obrero V, Diaz-Villanueva A, Sanz-Pamplona R (2022). Zenodo.

[40] Barbeira AN (2020). Fine‐mapping and QTL tissue‐sharing information improves the reliability of causal gene identification. Genetic Epidemiology.

[41] The GTEx Consortium (2020). The GTEx Consortium atlas of genetic regulatory effects across human tissues. Science.

[42] Solé X (2014). Discovery and Validation of New Potential Biomarkers for Early Detection of Colon Cancer. PLoS ONE.

[43] The Cancer Genome Atlas Research Network (2013). The Cancer Genome Atlas Pan-Cancer analysis project. Nat Genet.

[44] Díez-Villanueva A (2020). DNA methylation events in transcription factors and gene expression changes in colon cancer. Epigenomics.

[45] Ritchie ME (2015). limma powers differential expression analyses for RNA-sequencing and microarray studies. Nucleic Acids Res..

[46] Sanz-Pamplona R (2015). Exome Sequencing Reveals *AMER1* as a Frequently Mutated Gene in Colorectal Cancer. Clin Cancer Res.

[47] shiny: Web Application Framework for R. (2017).

[48] Stegle O, Parts L, Piipari M, Winn J, Durbin R (2012). Using probabilistic estimation of expression residuals (PEER) to obtain increased power and interpretability of gene expression analyses. Nat Protoc.

[49] Kozomara A, Griffiths-Jones S (2014). miRBase: annotating high confidence microRNAs using deep sequencing data. Nucl. Acids Res..

