# Author Correction: COLONOMICS - integrative omics data of one hundred paired normal-tumoral samples from colon cancer patients

**DOI:** 10.1038/s41597-022-01829-x

**Published:** 2022-11-18

**Authors:** Anna Díez-Villanueva, Rebeca Sanz-Pamplona, Xavier Solé, David Cordero, Marta Crous-Bou, Elisabet Guinó, Adriana Lopez-Doriga, Antoni Berenguer, Susanna Aussó, Laia Paré-Brunet, Mireia Obón-Santacana, Ferran Moratalla-Navarro, Ramon Salazar, Xavier Sanjuan, Cristina Santos, Sebastiano Biondo, Virginia Diez-Obrero, Ainhoa Garcia-Serrano, Maria Henar Alonso, Robert Carreras-Torres, Adria Closa, Víctor Moreno

**Affiliations:** 1 Oncology Data Analytics Program, Catalan Institute of Oncology (ICO). Hospitalet de Llobregat, Barcelona, Spain; 2 Colorectal Cancer Group, ONCOBELL, Bellvitge Biomedical Research Institute (IDIBELL). Hospitalet de Llobregat, Barcelona, Spain; 3 Biomedical Research Centre Network for Epidemiology and Public Health (CIBERESP), Madrid, Spain; 4 Molecular Biology CORE, Center for Biomedical Diagnostics, Hospital Clínic de Barcelona, 08036 Barcelona, Spain; 5 Translational Genomic and Targeted Therapeutics in Solid Tumors, August Pi i Sunyer Biomedical Research Institute (IDIBAPS), 08036 Barcelona, Spain; 6 Unit of Nutrition and Cancer, Cancer Epidemiology Research Program, Catalan Institute of Oncology (ICO) - Bellvitge Biomedical Research Institute (IDIBELL). L’Hospitalet de Llobregat, Barcelona, 08908 Spain; 7 Department of Epidemiology, Harvard T.H. Chan School of Public Health, Boston, MA 02115 USA; 8 Rheumatology Department - Parc Taulí Research and Innovation Institute (I3PT), Barcelona, Spain; 9 TIC Salut Social Foundation. Ministry of Health of Generalitat de Catalunya, Barcelona, Spain; 10 Reveal Genomics. S.L., Barcelona, Spain; 11 Department of Clinical Sciences, Faculty of Medicine and health Sciences and Universitat de Barcelona Institute of Complex Systems (UBICS), University of Barcelona, Barcelona, Spain; 12 Medical Oncology Department. Catalan Institute of Oncology (ICO), Hospitalet de Llobregat, Barcelona, Spain; 13 Biomedical Research Centre Network for Oncology (CIBERONC), Madrid, Spain; 14 Pathology Service, Bellvitge University Hospital (HUB), Hospitalet de Llobregat, Barcelona, Spain; 15 Digestive Surgery Service, Bellvitge University Hospital (HUB). Hospitalet de Llobregat, Barcelona, Spain; 16 The John Curtin School of Medical Research, Australian National University, Canberra, Australia; 17 EMBL Australia Partner Laboratory Network at the Australian National University, Canberra, Australia

**Keywords:** Cancer genomics, Biomarkers, Colon cancer, Cancer genomics, Biomarkers, Colon cancer

Correction to: *Scientific Data* 10.1038/s41597-022-01697-5, Article published online 1 October 2022

In this article affiliation details for Cristina Santos were incorrectly given as ‘Department of Clinical Sciences, Faculty of Medicine and health Sciences and Universitat de Barcelona Institute of Complex Systems (UBICS), University of Barcelona, Barcelona, Spain’; ‘Biomedical Research Centre Network for Oncology (CIBERONC), Madrid, Spain’ & ‘Pathology Service, Bellvitge University Hospital (HUB), Hospitalet de Llobregat, Barcelona, Spain’ but should have been ‘Colorectal Cancer Group, ONCOBELL, Bellvitge Biomedical Research Institute (IDIBELL). Hospitalet de Llobregat, Barcelona, Spain’; ‘Department of Clinical Sciences, Faculty of Medicine and health Sciences and Universitat de Barcelona Institute of Complex Systems (UBICS), University of Barcelona, Barcelona, Spain’; ‘Medical Oncology Department. Catalan Institute of Oncology (ICO), Hospitalet de Llobregat, Barcelona, Spain’ & ‘Biomedical Research Centre Network for Oncology (CIBERONC), Madrid, Spain’.

In addition, the affiliation ‘Colorectal Cancer Group, ONCOBELL, Bellvitge Biomedical Research Institute (IDIBELL). Hospitalet de Llobregat, Barcelona, Spain’ for Ramon Salazar was missing.

The original article has been corrected.

